# A Study on Acute Management of Colorectal Cancer Presenting as an Emergency

**DOI:** 10.7759/cureus.81591

**Published:** 2025-04-01

**Authors:** Ajit Singh Oberoi, Paul A Peters, Neil Muscat, Imran Alam, Chelliah R Selvasekar

**Affiliations:** 1 Surgery, Queen Elizabeth University Hospital, Glasgow, GBR; 2 General Surgery, Royal Albert Edward Infirmary, Wigan, GBR; 3 Colorectal Surgery, The Christie NHS Foundation Trust, Manchester, GBR

**Keywords:** colorectal cancer, colorectal cancer surgery, emergency surgery, palliative medicine, palliative stenting

## Abstract

Introduction

Many patients in Greater Manchester underwent emergency/urgent surgery, but limited data are available about managing these patients and their outcomes. We aimed to study the clinico-demographic profile, management trends, and outcomes of the patients presenting to the ED in the real world.

Methods

All patients with lower GI cancer (known or newly diagnosed on the present visit) in the ED requiring admission under surgeons for treatment were identified prospectively over six months and were included in the study.

Results

Nineteen patients were admitted (13 (69%) colon, 5 (26%) rectum, 1 (5%) anal cancer), with a median age of 67 years, and 10 (52.6%) were female. Ten (53%) patients were on the two-week wait pathway, and 14 (73.6%) patients presented with obstruction symptoms. Only 3 (15.7%) patients had received neoadjuvant therapy. Most had moderately differentiated adenocarcinoma. All had CT of the abdomen on admission, with 9 (52.6%) having CT of the thorax. All had locally advanced or metastatic cancers. Twelve (63%) patients underwent surgery (6 (50%) definitive, 6 (50%) diversion stoma). Nine (75%) patients had early postoperative complications, and 6 (50%) required ICU admission. The median postoperative stay was 9.5 days, with a 30-day mortality rate of 25%.

Conclusion

This study provides a snapshot of the management and outcomes of patients presenting in emergencies. A further study at the regional/national level is needed to investigate the increasing emergency presentations of colorectal cancer (CRC) despite an established screening program.

## Introduction

Colorectal cancer (CRC) ranks among the top three most common cancers in Greater Manchester [1]. Between 2017 and 2019, the United Kingdom (UK) recorded an average of 44,063 new cases and 16,808 deaths annually [2]. The COVID-19 pandemic caused delays in diagnosis and treatment, leading to an increased number of CRC patients presenting to EDs [3]. These patients often require interventions such as surgery, stenting, interventional radiology, or palliative care. The National Bowel Cancer Audit (NBOCA) 2022 reported that 27% of CRC patients in Greater Manchester underwent emergency or urgent surgery. In 2023, 19.1% (5,907) of CRC patients nationally were admitted urgently [1]. Despite this, there is limited data on how these patients are managed and how this impacts their prognosis and survival. Medical care in the UK is delivered through the National Health Service (NHS) and its trusts. In our trust, the on-call emergency consultant rota is shared among general surgeons and colorectal surgeons. Additionally, CRC management in emergency departments varies across trusts, influenced by factors such as the availability of specialists, out-of-hours gastroenterologists, interventional radiology facilities, and local policies.

This study aimed to profile the CRC patients presenting in an emergency, their treatment patterns, and outcomes among these patients in the real world at one of the district general hospitals in Greater Manchester.

## Materials and methods

A prospective service evaluation project was conducted over six months at a single center. The primary aim was to examine the clinico-demographic profile of patients presenting with colorectal and anal cancer in the ED, along with the management trends and real-world outcomes for these patients. The secondary aim was to inform future studies on the processes, management, and outcomes of emergency CRC care in Greater Manchester. The study included patients with known or newly diagnosed colorectal or anal cancer who required surgery, stenting, or palliative treatment. Exclusion criteria included readmissions, which were considered part of the initial admission's outcome, and admissions for non-surgical reasons such as chemotherapy-related complications or other medical issues.

Patients were identified from the daily handover sheets, which were updated at least twice a day. Data were collected using a proforma, based on clerking notes, operation notes, and daily review notes entered online by the reviewing physician. In this study, short-term outcomes were defined as those occurring within 30 days of surgery, while late outcomes were defined as those occurring between 30 and 90 days post-surgery.

Ethics

The study was registered with the hospital’s Quality Improvement and Audit Department as a service evaluation project. Since the data collected were part of routine practice, ethical approval from the NHS research was not required. As it involved evaluating services routinely provided to patients, additional informed written consent was not necessary. The proformas were completed as part of the standard procedure and stored securely in a locked location. The only patient-identifiable information recorded was the hospital number, used to track readmissions and long-term outcomes. Data from the proformas were entered into a coded Excel sheet, which was then disposed of in a confidential waste bin. The Excel sheet was stored on a password-protected trust computer.

Statistical analysis

Data were recorded in Microsoft Excel, which was used for statistical analysis. Quantitative variables such as age, duration of symptoms, hemoglobin levels, albumin levels, and postoperative stay were expressed as means/medians with ranges. Qualitative variables, including sex, family history of cancer, smoking, alcohol use, cancer location, receipt of neoadjuvant treatment, availability of preoperative scopes, CT, and MRI scans, Tumor, Node, and Metastasis (TNM) staging, and treatment patterns, were recorded as proportions. Various charts were used to visually represent the data.

## Results

Demography

A total of 19 patients participated in the study, with a median age of 67 years (IQR 23). Most patients lived with family, while 5 (26.5%) lived alone. Demographic information is summarized in Table 1.

**Table 1:** Demographic profile.

Variable	Results
Total number of patients	19
Median age at presentation (years)	67 (37-91)
Ethnicity	British white: 18
	Irish white: 1
Gender distribution (Male: Female)	9:10
Family history of colorectal cancer (n=8)	Yes: 25%
Smoking (n=17)	Yes: 41.2%
Alcohol (n=16)	Yes: 50%
House occupancy	Single: 5 (26.3%)
	Double: 14 (73.7%)
Site of tumour	Colon: 69%
	Rectum: 26%
	Anus: 5%

**Figure 1:**
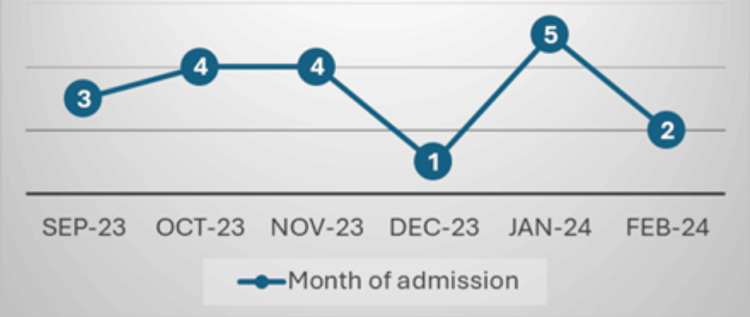
Admissions every month.

There was a monthly variation in patient admissions, with the highest number of admissions occurring in January (5 patients; 26.3%) and the lowest in December (1 patient; 5.2%) (Figure 1).

Of the patients, 13 (69%) had colonic cancer, with 10 (52%) diagnosed with left colon cancer and 3 (17%) with right colon cancer. Ten (53%) patients were referred through the 2-week wait (2 ww) pathway. Among these, 8 (42%) had already been discussed in a multidisciplinary team (MDT) meeting and started treatment, while 2 patients (11%) were still awaiting MDT discussion.

Clinical profile

The median duration of symptoms was 3 days (range 1-56 days). The most common presenting symptoms were obstruction (n=14; 73.6%) and perforation (n=2; 10.5%). Most patients had a WHO performance status of 3/4 (n=10; 52.7%), while the remaining 9 (47.3%) had a performance status of 0/1/2. Three patients (15.7%) received neoadjuvant therapy, including two who underwent chemoradiotherapy and one who received chemotherapy alone. All three patients completed their treatment. Among them, two (66.6%) experienced disease progression. Patients underwent various investigations including blood tests, endoscopies, CT scans, and MRI scans before or during the admission before intervention (Table 2).

**Table 2:** Investigations performed for patients. CEA: Carcinoembryonic Antigen.

Clinical Work-up	Values
Haemoglobin (median) (g/L)	130 (78-172)
Albumin (median) (g/L)	39 (23-56)
CEA (n=8)	50% raised
Preadmission scope available?	31.50%
CT Abdomen and pelvis available at admission?	100%
CT Thorax available at admission?	52.6% (9)
MRI Pelvis before admission	5/6 patients of anorectal cancer had MRI
Histology (n=17)	Adenocarcinoma: 15
	Squamous cell carcinoma: 1
	Lipomatous: 1

Twelve patients (63.1%) had hemoglobin <130 mg/dL, and 6 patients (31.55%) had hypoalbuminemia (albumin <35 mg/dL). A CT of the abdomen and pelvis was done in all the patients at admission, and 10 (52.6%) had a CT of the thorax at admission. Preadmission colonoscopy was available in 6 (31.5%) patients, and all had an ulcero-proliferative growth.

Most patients had advanced cancer, with all having either T3 (n=4; 22%) or T4 (n=15; 78%) disease. Only 5 (28%) patients had N0 disease, while 10 (50%) had N1 disease, and 4 (22%) had N2 disease. Forty-five percent of patients had metastatic disease, with the liver and bones being the most common sites of metastasis. Tumor histology data were available for 17 patients, of whom 15 (88.2%) had adenocarcinoma, while one had squamous cell carcinoma, and one had lipomatous histology. Most patients had moderately differentiated tumors (57.1%), while the remaining 42.9% had poorly differentiated tumors. Mutations were identified in six specimens, including BRAF (3), MLH1 (2), KRAS (2), PMS2 (1), and MMR (1).

Treatment patterns

Two-thirds of the patients (n=12, 63.1%) underwent surgical intervention, while one-third (n=7, 36.8%) received palliative/best supportive care. No patients underwent stenting. An equal number underwent definitive surgery (n=6, 50%) and diversion stoma (n=6, 50%). Definitive surgeries included segmental colectomies (right hemicolectomy or Hartmann procedure) and en-bloc resection for advanced tumors. Of the surgeries, 92% (n=11) were performed via an open approach, and 8% (n=1) via minimally invasive surgery (MIS). Most surgeries were conducted by general surgeons (n=7; 58%), with colorectal surgeons performing the remaining 42% (n=5).

Treatment outcomes of patients undergoing surgery

No intraoperative complications were reported in any of the patients (Table 3).

**Table 3:** Surgical outcomes.

Variables	Frequency
Intraoperative complication	0
Early postoperative complication (<30 d)	Total: 9 (75%)
	Cardiopulmonary: 6 (66.6%)
	Wound related: 3 (33.3%)
	Stoma related: 1 (11.1%)
	Others: 2 (22.2%)
Late postoperative complication (30-90 d)	0
Patients requiring ICU stay	6 (50%)
Median ICU stay	5.5 days (2-10)
30-day mortality	3 (25%)
Median postoperative stay	9.5 days (2-33)

Seventy-five percent (n=9) of those undergoing surgery experienced early postoperative complications, with cardiopulmonary issues (n=6, 66.6%) and wound-related complications (n=3, 33.3%) being the most common. No late postoperative complications were reported. Half of the patients (n=6) required an ICU stay, with a median ICU stay of 5.5 days (range 2-10 days). The 30-day mortality rate was 25%, with two-thirds of these deaths attributed to cardiopulmonary complications after the surgery. The median postoperative hospital stay was 9.5 days (range 2-33 days).

## Discussion

CRC is the third most common cancer and the second leading cause of cancer death globally [4]. In England and Wales, 35,779 CRC cases were diagnosed from April 2021 to March 2022 [1]. However, there is limited data on the management of CRC patients in emergency settings. This study aimed to explore the clinic-demographic profile, treatment patterns, and outcomes of CRC patients admitted through emergency services at a single center.

This study included 19 patients admitted via emergency services over a 6-month period. The median age at presentation was 67 years, with a slight predominance of females. The national average age for CRC presentation is between 60-74 years, and 29.1% of patients requiring emergency admissions fall within this age range [1]. Additionally, CRC is slightly more common in males than in females [1]. The findings of this study may be influenced by the small sample size. Lower socioeconomic status and living alone are known social determinants for CRC [5], and approximately a quarter of the patients in this study lived alone. It is hypothesized that patients without support at home may be less likely to seek screening or medical attention for their symptoms. A key aspect of CRC treatment, as per the 2-week wait pathway, involves discussion in a MDT. Eighty percent of patients (n=8) on the 2ww pathway had an MDT discussion, and 87.5% (n=7) of these patients were already receiving palliative chemotherapy or best supportive care.

Most patients in this cohort presented with a short history, and the most common presentation was bowel obstruction. The majority had left-sided tumors (left colon and rectum), which are typically ulceroproliferative lesions that lead to bleeding or obstruction. NBOCA data also suggests that nearly 50% of emergency admissions are due to tumors in the splenic flexure, descending colon, sigmoid, rectosigmoid, or rectum. WHO performance status is a key factor in determining treatment and prognosis for CRC patients [6,7]. In this study, 47.3% of patients had a performance status of 0/1/2, indicating that they were fit for elective treatment. These patients, if treated electively, would likely have undergone formal MDT discussions, treatment planning, and potentially better outcomes. However, most patients presenting in the ED were not optimized for systemic treatment or intervention. Specifically, 63.1% had a hemoglobin level <130 mg/dL, and 31.5% had hypoalbuminemia. Additionally, many patients lacked a full workup, as evidenced by the availability of CEA levels, preoperative endoscopes, and CT thorax in only 42.1%, 31.5%, and 52.6% of cases, respectively. However, most patients (83.3%) with anorectal cancer had an MRI available before admission. The most common imaging modality used in the emergency setting was a CT scan of the abdomen and pelvis, as it is readily available, provides a quick diagnosis, and aids in decision-making. CT helps identify the site of pathology, related complications such as bowel necrosis or perforation, and gross metastasis.

All patients in this study who presented in the ED had advanced cancers. NBOCA data suggest that 30.7% of patients requiring emergency admissions have metastasis [8]; however, this study found a higher incidence of metastasis at 45%. A study by Ahmadinejad M et al. also indicated that patients undergoing emergency surgery tend to have more advanced TNM stages and histologic grades [9]. In their study, over 59% of emergency surgery patients were in stages 3 or 4, compared to only 31% of those undergoing elective surgery. An advanced stage at presentation is a poor prognostic indicator, predicting lower survival rates. NHS data show that in England, patients with stage 3 or 4 bowel cancer have 5-year survival rates of 65% and 10%, respectively, significantly lower than the 90% and 85% survival rates for stage 1 and stage 2 cancers [10]. NBOCA data also highlight that 40.2% of screen-detected tumors are early-stage, offering a better prognosis [8], underscoring the importance of encouraging bowel cancer screening.

In this study, a majority of the cancers (42%) were poorly differentiated, which is also a poor prognostic marker. Of the 19 patients, 63% (n=12) underwent surgery, including both definitive tumor surgery and diversion stomas. The remaining patients were not deemed fit for surgery and were provided with best supportive care. No patients underwent stenting. Among those who had surgery, 8 had left colonic tumors, 2 had mid-rectal tumors, and 2 had ascending colon tumors. All surgeries, except one, were performed via an open approach. Factors limiting the use of minimally invasive surgery (MIS) in emergencies included patient fitness, the urgency of the surgery, surgeon expertise, and peritoneal soiling. While MIS is now the standard for elective CRC surgeries, its role in emergency settings remains debated. A retrospective cohort study by Alselaim NA et al. found better short-term postoperative outcomes with laparoscopic surgery for emergency colorectal cases [11]. A recent meta-analysis by Cirocchi on laparoscopic colectomy for right-sided colonic tumors also found improved short-term outcomes with no difference in overall survival [12]. However, these results should be interpreted with caution due to the limited evidence and a small number of studies. Larger studies, such as the multicenter randomized controlled trial LapEmerge, comparing laparoscopic versus open approaches in emergency colorectal surgery, are expected to provide more definitive evidence.

Studies have shown poorer postoperative outcomes following emergency colorectal surgeries compared to elective procedures [13-15]. It has been suggested that cardiopulmonary fitness impacts postoperative recovery, and that preoperative cardiopulmonary exercise testing can predict morbidity and mortality [16,17]. In this study, 75% of patients experienced early postoperative complications, including cardiopulmonary (66.6%), wound-related (33.3%), and stoma-related (11.1%) issues. The median postoperative stay was 9.5 days, with 50% of patients requiring an ICU stay, and the 30-day mortality rate was 25%. Similar outcomes have been reported in the literature. A retrospective cohort study by Amri R et al., which included 1,071 patients, compared the outcomes of emergency surgery for colon cancer with elective surgery [13]. They found higher perioperative mortality (7.8% vs 0.8%, p<0.001) and longer hospital stays (8% vs 5%, p<0.001) in emergency cases. Ahmadinejad M et al. also found worse outcomes in emergency surgery, with a median hospital stay of 29 days and a mortality rate of 20% for patients undergoing emergency surgery, significantly higher than for those who underwent elective surgery [9]. Data from the bi-national colorectal cancer database audit, which included 15,676 patients (13.6% emergency cases), also showed poorer outcomes for emergency surgeries, with surgical complications, medical complications, and mortality rates of 26.7%, 22.8%, and 3.4%, respectively, all significantly higher than for elective resections [15].

Poor surgical outcomes in emergency surgery are often due to the acute nature of the surgery, advanced cancer stage, poor physiological reserves, and limited expertise outside regular hours. Additionally, there is limited time for objective assessments of fitness, such as cardiopulmonary testing, or for preoperative rehabilitation. In this study, over 60% of patients had a hemoglobin level below 130 mg/dL, and over 30% were hypoalbuminemic, contributing to poor recovery. Furthermore, 92% of surgeries were performed via an open approach, which can also impact early postoperative outcomes [11]. None of the patients underwent stenting due to logistical challenges, limited availability of skilled endoscopists, and the unavailability of stenting services outside regular hours. Multiple studies have demonstrated that stenting as a bridge to definitive surgery can improve outcomes, particularly for left-sided tumors [18,19]. The ESCO trial also found that colonic stenting for left-sided obstructing tumors significantly reduced the rate of stoma formation [20]. In this study, 10 patients had left-sided colon or mid-rectal tumors, and 7 of them presented with obstruction. These patients could have been potential candidates for stenting, which may have led to better outcomes.

The strength of this study lies in its prospective design, providing real-world evidence on the management of these patients. Additionally, being a single-center study, it offers better internal validity. However, an important limitation is the small sample size, which means the results should be interpreted with caution when generalizing to the broader population. Moreover, there was missing data for several patients, including details on smoking history, house occupancy, and examination findings. The results of this study have been submitted to the regional cancer center, which has now approved a region-wide study. This larger study will help identify vulnerable patient populations, gaps in cancer screening, issues within the 2-week wait pathway, treatment patterns, and will guide future policies and resource allocation for patient management. It will also be valuable to assess long-term outcomes, including survival and quality of life, for patients undergoing emergency interventions.

## Conclusions

This study outlines the patient profiles, treatment patterns, outcomes, and gaps in managing colorectal cancer patients who present in an emergency. Most patients were elderly and not fully optimized for major interventions. They often presented with advanced tumors complicated by obstruction or perforation. Surgery was the main treatment at our center, but it was associated with notable postoperative complications and mortality. Only one patient underwent laparoscopic surgery, and none received stenting. Both interventions, if applied to appropriate patients, could have potentially improved outcomes.
